# Routine cine-CMR for assessment of prosthesis-associated mitral regurgitation - a multicenter, multivendor study

**DOI:** 10.1186/1532-429X-15-S1-E100

**Published:** 2013-01-30

**Authors:** Lauren A Simprini, Anika Afroz, Igor Klem, Christoph J Jensen, Raymond J Kim, John F Heitner, Michael Sood, Elizabeth Chandy, Dipan J Shah, Juan C Lopez-Mattei, Robert W Biederman, Anthon Fuisz, Kambiz Ghafourian, Jonathan W Weinsaft

**Affiliations:** 1 Medicine/Cardiology, Weill Cornell Medical College, New York, NY, USA; 2 Medicine/Cardiology, Memorial Sloan Kettering Cancer Center, New York, NY, USA; 3 Duke Cardiovascular Magnetic Resonance Center, Durham, NC, USA; 4 New York Methodist Hospital, Brooklyn, NY, USA; 5 The Methodist DeBakey Heart & Vascular Center, Houston, TX, USA; 6 Allegheny General Hospital, Pittsburg, PA, USA; 7 Drexel University College of Medicine, Philadelphia, PA, USA; 8 Washington Hospital Center, Washington, DC, USA

## Background

Mitral regurgitation (MR) is clinically important for patients with prosthetic mitral valves (PMV). While CMR can quantify MR based on flow, this requires dedicated imaging. Cine-CMR (SSFP) provides an alternative approach, whereby MR can be graded based on regurgitation-associated intervoxel dephasing. As cine-CMR is a standard component of nearly all exams, it could be used to screen for patients who warrant further quantitative imaging. Diagnostic performance of cine-CMR for prosthesis-associated MR has not been evaluated.

## Methods

Databases at 6 sites were queried for all patients with PMV in whom CMR and echocardiography were performed within 10 days. Cine-CMR images were retrieved and interpreted using a uniform protocol: MR was visually graded based solely on jet size (mild <1/3, moderate 1/3-2/3, severe >2/3) in relation to the left atrium. MR was graded in each long axis plane (2-, 3-, 4-chamber), with overall severity based on mean grade. Additional parameters included jet directionality, signal intensity (3-grade scale), and pulmonary vein flow reversal. Echocardiography (TTE, TEE) was used as a comparator for MR based on clinically reported data. Cine-CMR was interpreted blinded to patient history and TTE/TEE.

## Results

56 patients with PMV (70% mechanical, 30% bio) underwent cine-CMR and echo (TTE 70%, TEE 48%) within 2.5±2.6 days. Cine-CMR (1.5T, typical TR=3msec, TE=1msec, BW=977Hz/pixel) was performed using commercial scanners (Siemens 59%/GE 36%/Philips 5%). MR was present on cine-CMR in 77% of patients (mild 43%, moderate 14%, severe 20%), and varied in direction (central 88%, eccentric 12%). Patients with severe MR had higher prevalence of dense regurgitant jets (73% vs. 3%, p<0.001), more frequent pulmonary vein reversal (55% vs. 3%, p<0.001), and larger left atria (5.7±1.0cm vs. 4.7±1.4cm, p=0.03) than did those with lesser MR, but did not differ based on LVEF (53±14% vs. 49±15%, p=0.4). Compared to TEE, cine-CMR yielded excellent diagnostic accuracy (96%) for severe MR (Table); accuracy was also high (93%) when a broader TEE threshold (≥moderate MR) was applied. Among patients with TTE and cine-CMR, no patients had severe MR on TTE missed by cine-CMR, whereas TTE was discordant (lesser MR) in 83% (5/6) with severe MR on cine-CMR. Among those with all three tests (n=10), TEE-evidenced severe MR was diagnosed by TTE in 50% (1/2), whereas cine-CMR diagnosed severe MR in all (2/2) cases.

**Table 1:** Cine-CMR diagnostic performance for prosthesis-associated mitral regurgitation

	Sensitivity	Specificity	Accuracy	Positive predictive value	Negative predictive value
Severe MR	100% (6/6)	95% (20/21)	96% (26/27)	86% (6/7)	100% (20/20)

Substantial (≥moderate) MR	100% (9/9)	89% (16/18)	93% (25/27)	82% (9/11)	100% (16/16)

Calculations based on transesophageal echocardiography reference standard.

## Conclusions

Among a blinded multicenter cohort with PMV, cine-CMR yielded excellent diagnostic performance (accuracy=96%) for TEE-evidenced severe MR. Findings support use of cine-CMR for non-invasive assessment of prosthesis-associated MR.

## Funding

K23 HL 102249-01.

